# Mechanical Strain-Enabled Reconstitution of Dynamic Environment in Organ-on-a-Chip Platforms: A Review

**DOI:** 10.3390/mi12070765

**Published:** 2021-06-28

**Authors:** Qianbin Zhao, Tim Cole, Yuxin Zhang, Shi-Yang Tang

**Affiliations:** 1 School of Electrical and Electronic Engineering, Nanyang Technological University, Singapore 639798, Singapore; 2 Department of Electronic, Electrical and Systems Engineering, University of Birmingham, Edgbaston, Birmingham B15 2TT, UK; TXC991@student.bham.ac.uk (T.C.); YXZ048@student.bham.ac.uk (Y.Z.)

**Keywords:** organ-on-a-chip, microfluidics, mechanical strain, actuators

## Abstract

Organ-on-a-chip (OOC) uses the microfluidic 3D cell culture principle to reproduce organ- or tissue-level functionality at a small scale instead of replicating the entire human organ. This provides an alternative to animal models for drug development and environmental toxicology screening. In addition to the biomimetic 3D microarchitecture and cell–cell interactions, it has been demonstrated that mechanical stimuli such as shear stress and mechanical strain significantly influence cell behavior and their response to pharmaceuticals. Microfluidics is capable of precisely manipulating the fluid of a microenvironment within a 3D cell culture platform. As a result, many OOC prototypes leverage microfluidic technology to reproduce the mechanically dynamic microenvironment on-chip and achieve enhanced in vitro functional organ models. Unlike shear stress that can be readily generated and precisely controlled using commercial pumping systems, dynamic systems for generating proper levels of mechanical strains are more complicated, and often require miniaturization and specialized designs. As such, this review proposes to summarize innovative microfluidic OOC platforms utilizing mechanical actuators that induce deflection of cultured cells/tissues for replicating the dynamic microenvironment of human organs.

## 1. Introduction

Laboratory preclinical testing is an important step in the validation and evaluation of new drug candidates before further human clinical trials, and is expected to reduce the emergency medical events caused by adverse drug reactions [1]. Pharmaceutical drug testing traditionally depends on in vivo animal models or in vitro cell culture models to evaluate pharmacological and toxicological responses to a new agent. However, it is known that in vivo animal models are sometimes challenged to precisely predict human physiological responses in drug development (e.g., metabolism, transport and oral absorption of drugs, etc.) and may poorly mimic the tissue–drug interactions. Animal testing is also hindered by issues like expensive cost, high operation difficulty, long time consumption as well as ethical approval [2,3,4].

On the other hand, in vitro cell and tissue cultures for elaborating biomimetic models have been deeply developed in recent decades to provide reliable and usable alternatives for study in various fields, such as biological research, pharmacodynamics, pharmacokinetics, drug toxicity screening, and therapy monitoring [5]. The response results are very important as a critical factor for the determination of whether the drug is safe and effective to advance to the next human clinical trials [6]. However, these responses also do not directly reflect human physiological responses because of the defective reconstitution of complex human organs.

Conventionally, cells are cultured in a 2D format to grow as a flattened monolayer independently without cell competition in flasks or well plate devices, which greatly contributes to the nascent in vitro artificial tissue for physiological-related applications [6]. More recently, 3D cell culture methods have emerged and transcended the traditional 2D method with the significant advantage of providing the in vivo-like living organ microenvironment, which is necessary to cell differentiation and specific tissue function reproduction. Cells cultured in 3D normally exhibit improved morphology, differentiation level, cell function, viability, and physiological and mechanical properties [6]. Nonetheless, static 3D cell culture technologies still desire to re-establish and integrate the dynamic microenvironment of in vivo organs. As a result, the convergence of microfluidic technologies and traditional cell culture protocol give rise to the blooming development of 3D cell dynamic culture to realise the concept of organ-on-a-chip (OOC), offering a vast opportunity for providing an alternative to animal models in preclinical drug screening.

It is well known that mechanical inputs can trigger a variety of molecular events that modulate physio-chemical outputs in microphysiological systems [7,8]. Therefore, actuators that can stretch and compress play an important role in mimicking in vivo physiologies in OOC technology with dynamic cell culture environments. Consequently, a variety of microfluidic devices utilizing integrated flexible actuators have been introduced to induce active stresses (shear, tensile, or compressive) for recreating physiological microenvironments. In most microfluidic OOC devices, highly actuate pumping systems (commercial syringe, prismatic, or pressure pumps) can be readily used to provide a precise control over shear stress induced by laminar, pulsatile, or interstitial flows. However, systems for providing mechanical strains are more complicated and may require specialized designs to generate proper levels of stimuli to tissues. To this end, this review seeks to highlight exciting microfluidic OOC platforms that are stimulated by mechanical strains. We will first provide a general overview of OOC technology. Next, mechanical strain enabled platforms with the reconstitution of mechanically dynamic microenvironments for mimicking different human organs will be elaborated. Finally, the review offers a perspective on the opportunities and challenges in developing OOC actuators for yielding future clinical advances.

## 2. A General Overview of Organ-on-a-Chip

Compared with traditional 3D cell culture, microfluidic OOC devices have better recapitulation of in vivo organ/tissue microstructures, cell–cell interactions, and physiological microenvironments. Therefore, OOC can take advantage of additional supporting modules to build more physiologically relevant artificial organ models so that the organ/tissue functions, activities, and physiological responses can be simulated more precisely in vitro [9]. In general, a microfluidic OOC system consists of four main modules: (1) The biological cell module that originates from various selective cell sources for dealing with the reproduced organ functionalities; (2) the microelectromechanical systems (MEMS) that provide stimulation and allows the fabrication of customized in vivo-like biomimetic frameworks; (3) the microfluidic module that reproduces the organ microenvironment; and (4) the sensing module that can monitor device operation (flow rate, strains, temperature, etc.) and detect chemicals (biomarkers, metabolites, oxygen level, etc.) [10,11]. As an early example, in 2004, Shuler’s group first proposed a microfluidic-based OOC prototype—microscale cell culture analogue (μCCA) system—to predict human response in conjunction with a physiologically based pharmacokinetic (PBPK) model in clinical trials. They fabricated a four-chamber lung–liver–other tissue–fat in vitro model with mammalian cells cultured on a silicon chip, and interconnected chambers with recirculating tissue culture medium to re-establish the circulatory system and specific organ functions [12,13].

In vivo human organs and tissues are formed as a complex structure consisting of different kinds of cells to perform specific functions. Hence, it is necessary for the in vitro model to artificially reconstitute the specific structure according to the in vivo native tissue morphology [14,15]. Since the animal model has been proved to be incapable of representing human organ physiology, human cell sources are more ideal for the artificial organ components [16,17]. Microsystems of OOC employ a microfabrication approach to build physiologically relevant cell culture incubators, which can recapitulate the functional unit tissue with minimal scaffolds of target organ, thereby reducing cost and maturation period [18,19,20]. In addition, cellular compatibility characteristics of natural or advanced synthetic hydrogel material allow a prolonged culture period and good cell attachment [21,22,23].

Microfluidics is a technology for the precise manipulation of a small quantity (from picolitre to microlitre level) of fluid. Leveraging its ability to control fluid networks in the microscale, the initial task of microfluidic technology in OOC is to continuously and precisely perfuse fresh culture medium with nutrients and oxygen to cells or tissues and remove the in situ metabolic wastes. Additional capabilities of microfluidic platforms have been further explored to provide in situ mechanical stimuli to cultured tissues, such as compression and expansion [24]. Mechanical stimuli have been demonstrated to play important roles in the regulation of biological processes at the cellular and tissue level. Living cells can be activated by mechanically changing the physiological microenvironment to maintain specific tissue/organ functions [25,26,27]. For instance, mechanical loading can increase the cardiomyocyte hypertrophy 2.2-fold and enhance proliferation rates by 21% compared to the case without stress [28]. Moreover, exposing vascular cells with fluid shear stress and cyclic stretch in a microfluidic platform to mimic the haemodynamic microenvironment can provide a better model for the in vitro study of blood vessel biomechanics [29].

Microfluidic OOCs have been tailored to mimic in vivo conditions using various materials and structures. Shear stress can be reproduced by the inherent advantages of off-chip precise fluid control systems, while strains can be generated using on-chip actuators. Pneumatic generation of mechanical stimuli on cells is the most developed technology in OOC applications, and normally incorporates the actuation of thin membranes of polydimethylsiloxane (PDMS). With the advantages of biocompatibility, low price, and precise casting, PDMS is the most widely used material for the fabrication of microfluidic OOC platforms. The elasticity of PDMS material is adjustable by tuning the ratio between elastomer base and curing agent, providing an intrinsic advantage to PDMS membranes with the potential to mimic different human tissues and organs. It has been proved that the elasticity of a substrate can affect cellular behaviours such as migration and differentiation for some types of cells [30]. For example, prostate carcinoma cells show the trend to migrate towards the less stiff substrate, while epithelial cells are more likely to move towards the stiffer substrate [31,32]; moreover, a suitable elastic substrate can contribute to the maturation of neural stem cell-derived neurons [33]. However, PDMS also has limitations for OOC applications—it can absorb small molecules such as drugs and secreted metabolites, which supresses the interaction of cells with stimulating substance. To solve this problem, alternative materials such as polylactic acid [34], poly(methyl methacrylate) (PMMA) [35], and polystyrene [36] can be used for fabricating membranes; moreover, lipophilic coatings can be applied to PDMS material to effectively prevent the absorbing issue [37]. In addition to silicone-based elastomers, hydrogel materials also possess the intrinsic advantages of being beneficial for cell engineering by mimicking the mechanical and structural cues of the human in vivo environment, which is expected to improve cell adhesion, proliferation, differentiation, and viability [38,39].

The following sections elucidate a variety of OOC platforms leveraging dynamic mechanical stimulations of tissues with microfluidic actuators for mimicking human organs such as the heart, kidney, lung, gut, and vessel. We mainly focus on OOC platforms equipped with mechanical actuators for inducing strains, while comprehensive reviews on OOC devices using other types of stimulations (e.g., electrical, magnetic, chemical, shear, etc.) can be found elsewhere [9,40,41,42,43,44,45].

## 3. Heart-on-a-Chip

**Figure 1:**
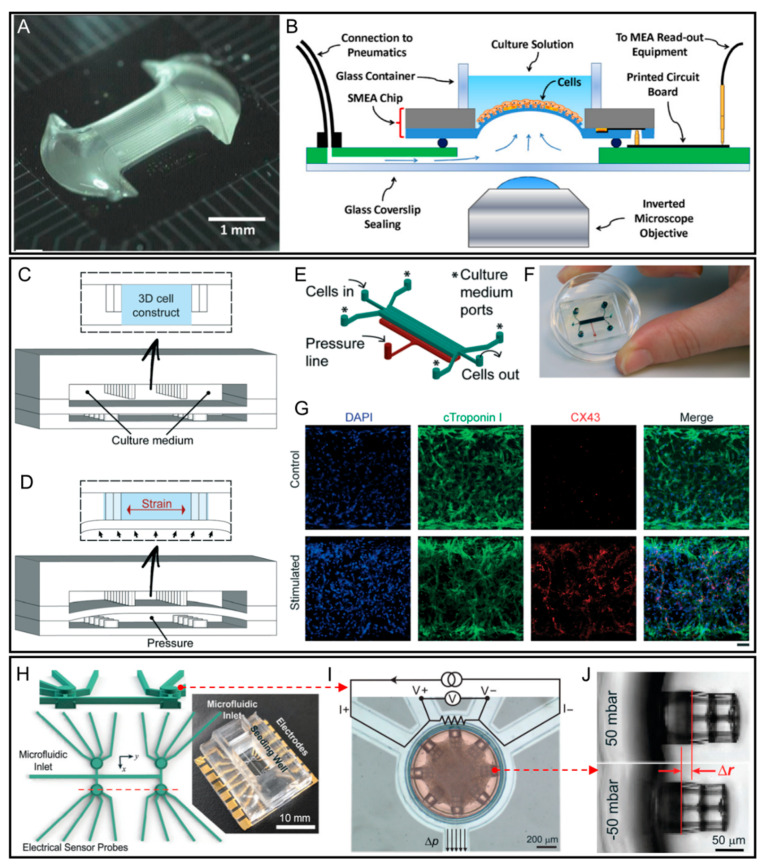
Heart-on-a-chip platforms with microfluidic actuators. (**A**) Photograph of the SMEA chip inflated with 15 kPa air pressure. (**B**) Schematic illustration of the SMEA chip system with a multi-layered configuration. Reproduced with permission from [48]. (**C**) Design of the 3D heart-on-a-chip microdevice proposed by Marsano et al. The device uses two compartmentalized PDMS microchambers separated by a PDMS membrane. (**D**) Deflection of the PDMS membrane by pressurizing the bottom compartment for producing a uniaxial strain to the 3D cell construct. (**E**) A 3D sketch of the channel design. (**F**) A picture of the actual heart-on-a-chip device. (**G**) Immunofluorescent images of specific cardiac markers such as cardiac troponin I (green) and gap junctions (connexin 43, red) of mechanically stimulated neonatal rat cardiac cells with or without stimulation (nuclei are stained by DAPI in blue), showing the effectiveness of cyclic mechanical strain on micro-cardiac construct maturation. Reproduced with permission from [50]. (**H**) Design and actual image of the platform fabricated using direct laser writing. (**I**) False-colored image showing a device with connecting microchannels: Seeding well with cardiac microtissue (red) and annular connecting microchannels (light blue). The top region of the annular microchannel acts as the electrical sensor. (**J**) Actuation of a cell-attachment site “cage” upon the application of pressure. Reproduced with permission from [52].

Cardiovascular diseases (CVDs) are the leading cause of death worldwide. While animal models have been widely used for the general understanding of the biological and physiological processes of CVDs, they often fail to exactly represent human cardiotoxicity due to inter-species differences [46,47]. For heart-on-a-chip systems, in addition to the electrical stimulation, mechanical contraction/expansion also plays a pivotal role in creating a precise model to mimic the main properties of the heart. To achieve this, Pakazad et al. presented a stretchable micro-electrode array (SMEA) chip for heart-on-a-chip application with a prolonged fatigue life and stable electromechanical characteristics for long-running cytostretch culturing [48]. As shown in Figure 1A, a PDMS membrane was sandwiched between a silicon substrate and sealed to act as a pneumatic channel. In stretching experiments, cells were seeded and adhered on the surface of the chemical treated PDMS membrane. The membrane was actuated by applying cyclic pressure within the backside pneumatic channel to realise cytostretch functionality (Figure 1B). In addition, the same group further developed a modularized membrane cytostretch platform based on similar microfabrication technology, which allowed rapid, low-cost, and mass production of different OOC applications [49].

Using an improved pneumatic microfluidic actuator, the study by Marsano et al. assessed the effects of the application of physiological cyclic uniaxial strains on the maturation and functionality of microengineered cardiac tissues (μECTs) generated by neonatal rat or human-induced pluripotent stem cell-derived cardiomyocytes (hiPSC-CM) (Figure 1C–G) [50]. This platform attempted to recapitulate the physiologic mechanical environment experienced by cells in the native myocardium. Cell-laden gels were confined between an array of hanging posts, and the actuation was pneumatically induced to generate homogeneous uniaxial cyclic strains to the cell constructs during culture (Figure 1B). The results demonstrated that the cyclic strain can be effectively and uniformly transferred to cells in culture, thereby increasing junction complexes and stimulating μECTs to realize superior cardiac differentiation and electro-mechanical coupling (Figure 1G). To solve the inherent shortcoming of mass production and break the barrier for commercial adoption, Agarwal et al. presented a high throughput integrated heart-on-a-chip device using MTF (muscular thin film) technology to realize the biomimetic diastolic and systolic stresses of the human heart [51]. The result of positive isoproterenol dose response studies proved the heart-on-a-chip system’s ability to simultaneously measure the contractile function during pharmacological interventions.

In a more recent study, instead of using conventional approaches, Jayne et al. used direct laser writing (DLW) lithography together with soft lithography to fabricate a platform for engineering cardiac microtissues in highly controlled microenvironments (Figure 1H) [52]. The platform contains four separate devices to house cardiac microtissues, and each individual device is equipped with an integrated strain actuator and a force sensor (Figure 1I). Each device also has a cell seeding well which is surrounded by an annular microfluidic channel. Cell attachment structures in the device can be actuated with the application of external pressure waves (Figure 1J). As such, the platform is able to induce time-dependent forces on cardiac microtissues derived from hiPSC. Conversely, oscillatory forces generated by the cardiac microtissues can be transduced into measurable electrical signals. This device possesses the potential to perform tissue characterization tasks and drug administration under applied strain, allowing for further studies of cardiac mechano-electric coupling.

## 4. Kidney-on-a-Chip

**Figure 2:**
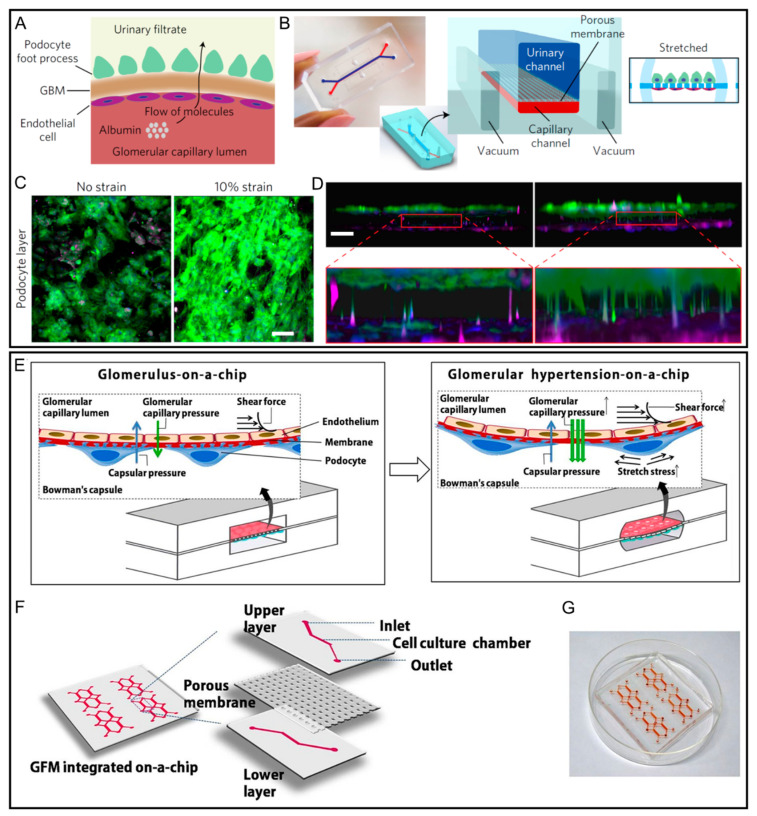
Kidney-on-a-chip platforms with microfluidic actuators. (**A**) Schematic representation of glomerular capillary wall with podocytes and endothelial cells separated by the glomerular basement membrane. An arrow shows directional flow of molecules from the capillary lumen to urinary space. (**B**) Photograph and schematic of the microfluidic organ-on-a-chip device with microchannels replicating the urinary and capillary compartments of the glomerulus. (**C**) Immunofluorescence microscopy images of podocytes differentiated in the device with or without a 10% mechanical strain. (**D**) Reconstructed side view of the tissue–tissue interface formed by hiPS-cell-derived podocytes (top, green) and human glomerular endothelial cells (bottom, magenta) showing that cyclic application of strain enhanced the extension of podocyte cell processes through the pores of the PDMS membrane. Scale bars are 100 μm. Reproduced with permission from [57]. (**E**–**G**) Schematics and image of the glomerulus-on-chip device. Mice glomerular endothelial cells (GEnCs) and mice podocytes (MPC-5) are cultured to confluence monolayers on both sides of the PDMS membrane for mimicking the glomerular filtration barrier. Perfused flow within the microchannel can induce shear stress, glomerular capillary pressure, and a stretching stress to reproduce glomerular hemodynamics in vitro. Reproduced with permission from [58].

Kidneys are the blood filter of our body which are responsible for controlling and modulating various body fluid activities, as well as drug excretion [53]. Kidney toxicity is one of the frequent causes of drug attrition and failure in drug development. Some serious adverse effects are observed after clinical testing stages due to the poor prediction ability of current preclinical drug screening technologies [46]. As a result, there is a need to reproduce an in vitro kidney function model to remedy the inaccuracy in conventional cell culture methods and animal models. Extensive research has shown that the introduction of shear stress using artificial tubular flow is expected to promote the development of artificial kidney-on-a-chip in the aspects of kidney dynamic microenvironment reconstitution [54,55,56]. In addition to shear, the modelling of mechanical strain is also necessary to mimic the cyclic pulsations of renal blood flow in living glomeruli. The work by Musah et al. utilized microfluidic organ-on-a-chip technology to model the human proximal tubule (Figure 2A–D) [57]. Terminally differentiated podocytes from human iPSCs were cocultured with endothelial cells on opposite sides of a laminin-coated PDMS porous membrane (Figure 2B). The separated microfluidic channels represent urinary and blood flow, while cyclic mechanical strain was applied to cell layers by stretching the flexible membrane using vacuum. Physiological differential clearance of albumin and inulin enabled tissue–tissue interface and the production of basement membrane collagen. The OOC platform also allowed for mimicking the toxicity of Adriamycin in vivo for causing podocyte disruption, loss of function, and cell death.

Furthermore, Zhou et al. presented a glomerulus-on-a-chip using a microchannel with a PDMS membrane (Figure 2E–G) [58]. The channel has a reconstituted pivotal glomerulus structure to mimic the glomerulus microenvironment, which was introduced to study glomerular hypertensive nephropathy as a nephron disease model. When injecting the culture medium from the upper inlet with an increasing flow rate (5, 10, and 15 μL/min) for inducing different glomerular hydrodynamic conditions, an increase in the permeability of medium-molecular weight (MW) and large-MW materials was observed. Here, glomerular hemodynamics was referred to mechanical stimulating forces induced by filtration pressure, shear stress, and mechanical strain. The results indicate that mechanical stimuli of hemodynamics could induce injury of cytoskeletal proteins and cell tight junctions, which further leads to the glomerular leakage of hypertensive nephropathy.

## 5. Lung-on-a-Chip

Lungs are the major respiratory organ of humans in the lower respiratory tract system, which are responsible for extracting oxygen from atmospheric air and excreting produced carbon dioxide to the outside of the body. The basic functional unit of the lung in respiration is the alveoli. Gas exchange takes place across the alveolar–capillary membrane, which is composed of a monolayer of epithelial and endothelial cells.

Humayun et al. presented a 3D thermoplastic-based human lung airway tissue model with suspended hydrogel to study the communication and interaction of airway smooth muscle cells (SMCs), airway epithelial cells (ECs), and the supporting extracellular matrix (ECM) [59]. The EC-SMC co-culture model was designed to be exposed to air for achieving biomimetic air–liquid interface culture; moreover, the model could be maintained with long-term viability for a prolonged period of more than one month. This lung-on-a-chip device is demonstrated to be suitable for mass production and possesses great potential to advance the research development in chronic lung diseases. The lung is a typical organ which involves mechanical motion at every moment. With the intrapleural pressure decreasing, the alveolar membrane is stretched with the expansion of alveoli after sucking air in. To mimic this process, Douville et al. introduced a microfluidic alveolar platform with cultured human epithelial cells to investigate ventilator-induced lung injury caused by fluid and mechanical stresses [60]. This study found that surface tension force was the dominant reason for cell death and detachment.

**Figure 3:**
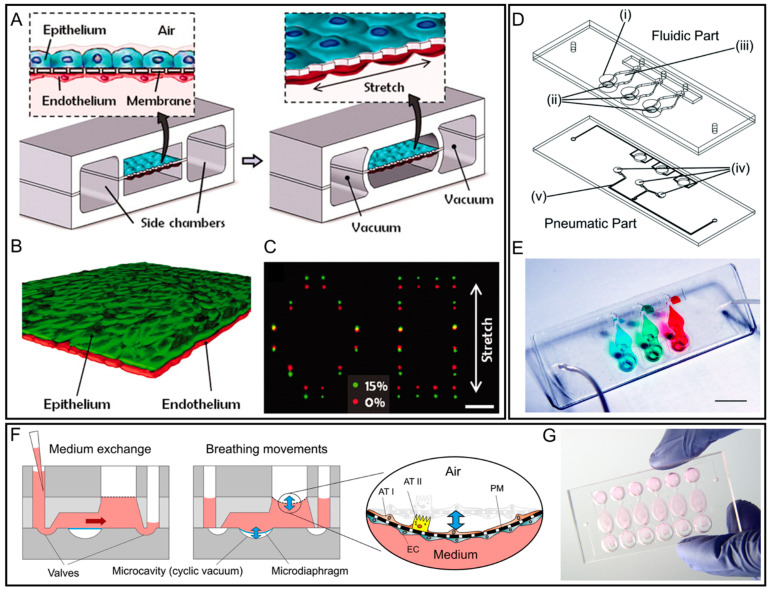
Lung-on-a-chip platforms with microfluidic actuators. (**A**) Schematic of reconstituted dynamic human lung-on-a-chip system. To mimic the similar in vivo alveolar–capillary microenvironment, air and fluid are introduced to the upper and lower microchannels isolated by a porous PDMS membrane. The membrane is sealed by monolayers of alveolar epithelial and microvascular endothelial cells. Reproduced with permission from [61]. (**B**) Long-term microfluidic coculture produces a tissue–tissue interface consisting of a single layer of the alveolar epithelium (Green) closely opposed to a monolayer of the microvascular endothelium (Red). (**C**) Membrane stretching-induced mechanical strain visualised by the displacements of fluorescent quantum dots. (**D**) Schematic and (**E**) actual image of a lung-on-a-chip system that is composed of a fluidic part and pneumatic parts. Primary human pulmonary alveolar epithelial cells are seeded onto the sandwiched PDMS membrane in the fluidic part, and the pressure from the micro-diaphragm beneath can be transmitted through the chamber with incompressible medium to the membrane. Reproduced with permission from [63]. (**F**) Schematic cross-sections of the alveolus-on-chip platform with two operation modes: Breathing and medium exchange modes. The breathing motions of the alveolar barrier are induced by a microdiaphragm when applying a cyclic vacuum to the microcavity. Hydrostatic and surface tension forces transport the flow. (**G**) Photograph of the chip with six independent alveolar barrier systems filled with cell culture medium. Reproduced with permission from [64].

Huh et al. presented a pneumatically activated microfluidic lung-on-a-chip system to re-establish the critical structural, functional, and mechanical features of the human alveolar–capillary interface. This work also demonstrated the recapitulation of complex human alveoli responses to bacteria and inflammatory cytokines [61]. This platform is based on a novel bioinspired microfluidic device that consists of a pair of vertical culture microchannels and two lateral pressure-driven microchannels (Figure 3A). Two culture microchannels were closely apposed and separated by a flexible porous membrane coated with extracellular matrix (ECM) gels, where human alveolar epithelial cells and pulmonary microvascular endothelial cells were seeded on the upper and lower surface, respectively, to form intact confluent monolayer tissue with the alveolar–capillary barrier function (Figure 3B). The pressure-driven microchannels were connected to a computer-controlled vacuum device to induce reversible expansion of adjacent culture channels. Simultaneously, cyclic elastic deformation of the culture channels could induce the unidirectional and uniform stretching and relaxing of the cultured tissue-to-tissue interface to mimic effects of dynamic mechanical strain (0.2 Hz, 10% strain) of the alveoli in respiratory movements (Figure 3C). Cells could maintain good viability for more than two weeks in the microchannel. Moreover, with the presence of the dynamic physiological environment, monolayer barrier function was improved with a higher trans-bilayer electrical resistance. It was also found that this lung-on-a-chip system performed an immune response to microbial infection similar to human in vivo lung alveoli. In addition, the cellular nanoparticle uptake ability of the alveolar epithelium and underlying endothelium was improved by the application of a cyclic mechanical strain. This pneumatically activated microfluidic system is not restricted to the simulation of human lung; it also offers the potential to develop other artificial organ models with the desired function of membrane-based penetration and mechanical stimuli. Furthermore, using the similar pneumatically actuatable human lung-on-a-chip model, Bai et al. studied the effect of the mechanical force generated by respiratory motion on the host innate immunity and investigated the lung alveolus model’s responses against viral infection [62]. When influenza H3N2 virus infected the in vitro lung alveolus model, the physiological breathing force could activate pulmonary epithelial and endothelial cells’ protective innate immune responses for increasing the number of circulating immune cells as occurred during viral infection in vivo. Besides, it is found that the hyper-physiological mechanical strain (10%) would enhance the endothelial cells’ inflammatory response. The proposed controllable lung-on-a-chip platform demonstrated the value for establishing therapeutic strategies to mitigate ventilator-associated lung injury.

Alternatively, a simplified and robust actuation using a bioinspired electro-pneumatic micro-diaphragm can be achieved for providing cyclic mechanical strain stimuli to build an easy-to-use lung-on-a-chip system (Figure 3D,E) [63]. While the system mentioned above is versatile and has been demonstrated to be competent in reconstituting alveolar–capillary interfaces for drug screening applications, the popularization of the pneumatic actuation protocol is hindered by the requirement of precise pressure control and a thin stretchable PDMS membrane with good elastic characteristics. An electro-pneumatic device was connected to the lung-on-a-chip system to apply a cyclic stretch. Compared with the static culturing condition, biomimetic respiratory movement significantly enhances the barrier permeability and metabolic activity function of human primary lung cells.

To realize additional functions, Stucki et al. created a ‘breathing’ lung-on-a-chip array, which is also capable of passive medium exchange. The chip includes a porous membrane (where cells are cultured on top), a microdiaphragm, and two valves (Figure 3F,G) [64]. The chip has two modes of operation—a breathing mode and a medium exchange mode. In the breathing mode, the two valves are shut, and the porous membrane is deflected when the microdiaphragm is moved by an applied negative pressure (Figure 3F). In the medium exchange mode, no ‘breathing’ takes place, and the valves are opened. Fresh medium can be inserted in the inlet, and the medium is exchanged passively within the chambers. The valves are then closed and a sample of the supernatant can be taken at the outlet. The chip was used to successfully culture human primary alveolar types I and II like epithelial cells over multiple days. Using a similar platform, the same group further examined the influence of mechanical strain on alveolar epithelial wound healing [65]. Furthermore, instead of using conventional PDMS membrane, the same group developed a model of alveolar air–tissue interface on a chip consisting of an array of suspended hexagonal monolayers of nanofibers (made from gelatin or collagen + elastin) supported by microframes [66,67]. The membrane was integrated into a microfluidic device for the patch integration. This membrane may outperform PDMS as it does not absorb rhodamine-B and is biodegradable; also, its thickness, composition, and stiffness can be readily tuned.

## 6. Gut-on-a-Chip

The gut is the primary digestive organ responsible for the absorption and digestion of nutrients, drugs, and minerals. Meanwhile, it is also the primary site for different kinds of diseases such as inflammation and infection. This leads to the need for drug testing using reliable in vitro human gut models that can substitute for animal models [68]. The intestinal functional unit is the human intestinal mucosa that serves as the barrier to the external world, where all physiological activities (transport, absorption, and protection, etc.) take place. The implementation and advances of microfluidic devices have greatly facilitated the development of in vitro human gut models, thereby showing great potential in replacing traditional methods (e.g., tranwell method) and animal models by providing more accurate bionic functions [69,70].

**Figure 4:**
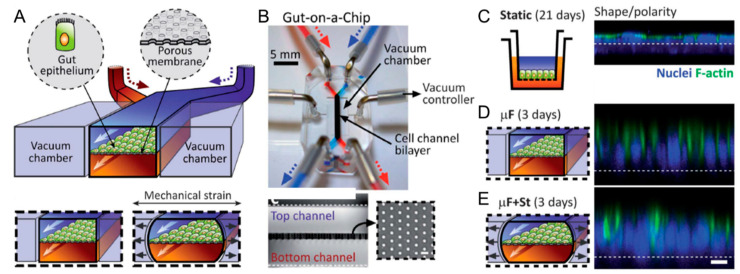
A gut-on-a-chip platform with microfluidic actuators. (**A**) Schematics and (**B**) images of the membrane-based gut-on-a-chip microdevice with pneumatic actuators. On the platform, the cultured Caco-2 cell monolayer is exposed to the low-level fluid flow (perfused from microchannel inlets) and cyclic biomimetic peristaltic motion (reproduced by pneumatically activated vacuum chambers). Morphology of the Caco-2 epithelial cells (**C**) cultured in the static transwell system and in the gut-on-a-chip with microfluidic flow (**D**) without or (**E**) with the application of cyclic mechanical strain (10%; 0.15 Hz) for three days. The confocal fluorescence views show a vertical cross section of the epithelium highlighting cell shape and polarity (nuclei in blue and F-actin in green). Scale bar is 20 μm. Reproduced with permission from [75].

In addition to widely reported desirable effects caused by shear or luminal flow in gut-on-a-chip systems [71,72,73], it has been demonstrated that a cyclic strain with physiologically relevant frequency and magnitude can also enhance proliferation and differentiation of human intestinal epithelial cells [74]. By utilizing external mechanical actuators, the muscle contractions or peristaltic flow can be accurately mimicked in gut-on-a-chip systems. To show this, Kim et al. presented a dynamic gut-on-a-chip system to reconstitute the human intestinal structure and physiology by reproducing the human gut’s dynamic intestinal peristaltic motion and flow (Figure 4) [75]. The microfluidic chip is similar to the above-mentioned lung-on-a-chip device (Figure 3A). The presence of dynamic physiological cues induces fluid shear stress (0.02 dyne/cm^2^) and cyclic mechanical strain (10% and 0.15 Hz) to significantly improve the epithelial cell proliferation, differentiation, and monolayer barrier function. The 3D intestinal villi-structured tissue was observed to be developed from epithelial cells (Caco2) that polarized rapidly and grew into undulations and folds. Moreover, the gut-on-a-chip system has great potential to benefit host–microbe symbiosis and evolution studies. It has been demonstrated that the artificial human gut model was able to support the co-culture of the normal intestinal microbe, *Lactobacillus rhamnosus* GG, and cultured epithelium monolayer for a prolonged time, over one week, without compromising the viability. Hence, this gut-on-a-chip system can recapitulate the fully functional epithelium monolayer tissue by leveraging the dynamic microenvironment for different studies on the in vitro intestinal model. In fact, the parallel vacuum chamber structure-induced mechanical stretch has been widely used in follow-up gut-on-a-chip and intestine-on-a-chip works [76,77].

While the in vitro artificial gut model was not established from primary cells or stem cells, it has been demonstrated by the same group that the poorly differentiated cell lines could still be qualified to undergo spontaneous villus morphogenesis, elevate mucus production, and improve drug metabolizing activity [78]. In the follow-up research on developing the gut-on-a-chip system, Kasendra et al. combined the revised pneumatic platform and organoid-based method to establish a primary human small intestine-on-a-chip device [79]. Primary intestinal epithelial cells that were isolated from individual patient biopsy-derived organoids were seeded and cultured on a PDMS membrane in the presence of human intestinal microvascular endothelial cells. After a 12-day dynamic culturing in a recapitulated mechanical microenvironment, epithelial cells were polarized and differentiated to form a 3D villus-like structure. On the other hand, the analysis of collected fluid samples demonstrated that the specific functions of digestion, nutrient response, and host response to infection revealed by the microfluidic organ model were closer to the living duodenum characteristics. Furthermore, Firoozinezhad et al. extended the in vitro co-culture experiment to human intestinal epithelial cells and aerobic human gut microbiota for studying the host–microbiome interactions [80]. Ingber’s group used the same chip to co-culture multiple commensal microbes to investigate the effects of microbiome, inflammatory cells, and mechanical peristaltic deformation on intestinal bacterial overgrowth and inflammation [81]. They claimed that bacteria would overgrow without the peristaltic strain, which was similar to the observation in patients with ileus and inflammatory bowel disease. More recently, Nelson et al. succeed in demonstrating the predictive ability of the human gut-on-a-chip platform for the characterization of an engineered live bacterial therapeutic [77]. Single bolus application of a synthetic live biotherapeutic, SYN5183 was applied to the gut compartment, resulting in dose-dependent increases in the biomarker, trans-cinnamic acid (TCA), and a corresponding 26.9% decrease in systemic Phe. The results showed a high degree of correlation with previously published non-human primate results.

## 7. Vessel-on-a-Chip

The blood vessel is the important circulatory organ spreading throughout our bodies. Nearly all other organs rely on the transport functionality of blood vessels as they can transport the nutrients, oxygen, and hormones to each organ and remove the in situ produced waste and carbon dioxide [82]. It is known that cardiovascular diseases are associated with the pathological changes of a blood vessel’s morphology and functions [83]. The microenvironment of the human vascular system provides complex mechanical stimuli to the tissues and cells due to the pulsatile blood flow and haemodynamic forces. For example, smooth muscle cells are the blood vessel’s major components. They are cyclically stretched and compressed by the pulsatile nature of blood flow. On the other hand, endothelial cells are also continuously exposed to the in vivo cyclic strain and shear stress [84]. Microfluidic OOC application with designed vascular functions can provide new sights into pathophysiology, facilitating the development of drugs and research of vessel physiology. While most literature focuses on the study of shear stress on the functionality of in vitro vessel models, much less attention has been paid to the effects cause by mechanical strains.

**Figure 5:**
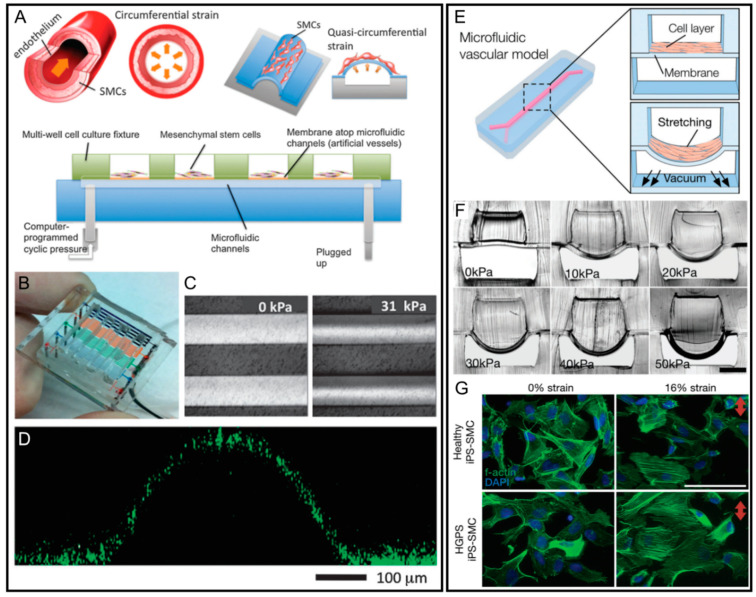
Vessel-on-a-chip platforms with microfluidic actuators. (**A**) Schematic illustration and (**B**) photography of the microfluidic artificial vessel chip. (**C**) The corresponding membrane deformations of 200 μm wide microchannel under the hydrostatic pressure from 0 to 31 kPa. (**D**) Confocal fluorescent cross-sectional image of deformed PDMS membrane on the 500 μm microchannel with the application of 21 kPa static pressure. Reproduced with permission from [86]. (**E**) Schematics of the pressure-controlled microfluidic vascular model and the biomimetic pulsatile blood flow strain effects. (**F**) Photographic images of microchannel cross-section deformation under different vacuum pressures inside the underlying air channel. (**G**) Different morphologies of HGPS iPS-SMCs and iPS-SMCs under a 16% strain treatment for 24 h. Reproduced with permission from [87].

To address this issue, a 3D microfluidic vascular chip was derived from the lung-on-a-chip design and was used to investigate the signalling between co-cultured human aortic endothelial cells (ECs) and aortic vascular smooth muscle cells (VSMCs) under the reproduced mechanical vascular microenvironment [85]. Using a different design, Zhou et al. proposed an in vitro microfluidic artificial vessel-on-a-chip platform that provided the biomimetic mechanically dynamic vascular microenvironment including the cyclic stimulation and circumferential strain via hydrodynamic actuation of microfluidic flow (Figure 5A,B) [86]. There was an array of straight microchannels with a width from 20 to 500 μm on the platform to mimic different-sized blood vessels, and each of the microchannels was covered by the suspended 35 μm thick PDMS membrane. They cultured human mesenchymal stem cells (MSCs) on the coated PDMS. Then, a computer-controlled hydrostatic pressure was applied to the liquid-filled microchannel, which contributed to the inflation of the elastic membrane, while the membrane returned to its original shape after releasing the pressure (Figure 5C,D). As a result, the in vivo vascular cyclic deformation due to hemodynamic pressure in the human artery was reproduced. It was observed that the dynamic platform could support MSCs to be cultured with good viability upon the application of a continuous cyclic strain for a prolonged period of more than seven days. The localization and alignment of MSCs generated by applied cyclic quasi-circumferential strain were observed. Signal transduction analysis of proteins can also be conducted on the platform.

Furthermore, Ribas et al. developed a pneumatic controlled progeria-on-a-chip platform to study the relationship between biomechanical train and vascular aging (Figure 5E–G) [87]. It is known that Hutchinson–Gilford progeria syndrome (HGPS) induces the accelerated aging of primarily vascular cells that experience mechanical force in vivo. The progeria-on-a-chip application was based on a stacked double-layered microchannel with two adjacent channels isolated by a PDMS membrane (Figure 5E). Smooth muscle cells (SMCs) were seeded onto the upper surface of the membrane and the upper channel was filled with culture medium. The underlying vacuum channel offered changing air pressure to generate cyclic deformation of the PDMS membrane, which reconstituted the biomechanical microenvironment of human blood vessels. The cyclic mechanical stretch varied from 5% to 25% to mimic the health physiological strain (5–10%) and pathological strain (>15%). It is shown that SMCs exposed to biomimetic strain would reorient perpendicular to the direction of strain and the aspect ratio of cell morphology increased with the increase in magnitude of strain. Moreover, if the strain magnitude increased to the pathological level, it induced similar mRNA expression profiles to the cells treated by angiotensin II. When the primary SMCs derived from human-induced pluripotent stem cells of HGPS donors (HGPS iPS-SMCs) and healthy donors were cultured into the proposed vessel platform, HGPS iPS-SMCs were observed to have an exacerbated inflammatory response to the applied strain (Figure 5G). This progeria-on-a-chip has great potential to offer an in vitro physically relevant disease model to study the vascular disease mechanism. Furthermore, using a similar setup, Jin et al. integrated a flexible and stretchable electrochemical sensor into a pneumatic microfluidic vessel-on-a chip platform to monitor the real-time biochemical signals induced by vascular mechanotransduction effects [88].

However, the above-mentioned vessel-on-a-chip platforms with pneumatic actuators require bulky external equipment for functionality; moreover, the planar geometry of microfluidic channels fail to mimic the native substrate and realistic geometry of the human vessel. To overcome these limitations, Dessalles et al. reported a luminal flow-actuated microvessel-on-a-chip system to reproduce the physiologically relevant levels of shear stress and strain exerted on the vessel wall without the requirement of external actuation [89]. In the collagen hydrogel-based cylinder microchannel (~100 μm in diameter), the coupled flow shear stress and circumferential strain were adjusted by the infused luminal pressure based on the poroelastic nature of hydrogel. The experiment indicated that the barrier effect to fluid and elasticity of endothelial monolayer could amplify the strain in hydrogel. This flow-induced actuating strategy provides an alternative actuation solution to microfluidic OOC platform, which promises a less complex constructure and more physiologically relevant mechanotransduction implementation.

## 8. Other Organ-on-a-Chip Platforms

In addition to typical organ models discussed above, OOC devices integrated with mechanical strain stimulation also enable the establishment of other highly reprinted functional artificial organs and specific disease screening models. For example, as the alternative for animal models, eye-on-a-chip is expected to reconstitute human eye physiology and anatomy for developing therapeutic strategies of human eye diseases such as dry-eye syndromes and macular degeneration [90]. Recently, Seo et al. proposed a reverse engineering eye-on-a-chip system using human corneal and conjunctional cells within a biomimetic physiological culture microenvironment [91]. They used an electromechanical actuator to reproduce the blink-induced cyclic mechanical forces at a frequency of 0.2 Hz, which is similar to the kinematics of spontaneous blinking of real human eyes. This customized ocular device could serve as the in vitro model of evaporative dry-eye disease for drug screening studies. As for modelling other diseases, Dolle et al. introduced a brain diffuse axonal injury model to study the axonal response to mechanical strain injury using a brain-on-a-chip system integrated with pneumatic actuators [92]. Moreover, to study the mechanical and physiological responses of the splenon, Rigat-Brugarolas et al. designed a functional microengineered red splenic pulp model, the minimal functional unit of spleen, for mimicking the in vivo hydrodynamic forces and physical properties of the human spleen [93].

## 9. Discussion and Conclusions

In this review, we summarized mechanical strain-enabled microfluidic OOC applications that reproduce the in vivo microenvironment based on living human organs. From the mentioned applications of heart/kidney/lung/gut/vessel-on-a-chip, various mechanical stimuli were mimicked by microfluidic strategies using pneumatic microchannels and elastomer membranes. It is observed that the applied mechanical stimuli can significantly improve the cell differentiation, proliferation, viability, tissue morphology, organ specific functions, and metabolizing activities. Apart from strain, normal hydraulic compression can also be induced using a similar pneumatic setups to stimulate bone tissues [94]. In conclusion, the mechanically dynamic in vivo microenvironment has an unneglectable improving effect on in vitro artificial human organ system establishment. This promises that the OOC system has the potential to be translated from lab-based prototypes to commercial products and replace current animal models in the future.

The pneumatic actuation approach is the most popular and versatile strategy to reproduce mechanical strain on chip, used together with suitably designed flexible elastomer membrane and shaped microchannels. However, the pneumatic actuation technology can impede the integration of OOC microfluidic systems due to the requirement of additional bulky pressure supplies, and micropumps for precise fluid flow operation. Moreover, the operation of OOC with actuators usually requires the installation of customized computer-controlled platforms, and this often involves a rather complicated system debugging process, which further complicates the adoption of OOC for researchers without engineering backgrounds. There is therefore the requirement for innovative actuation technologies, with the characteristics of: Easy operation/installation, easy control, low cost, relatively high accuracy, good integrity, and small volume. Some potential alternative actuation technologies include valveless electromagnetic micropumps, magnetorheological elastomers, and hydrogels.

Valveless electromagnetic micropumps with the nozzle/diffuser structure replacing valves can be used to simplify the pump structure so that it could be integrated onto the OOC chip [95]. The micropump could be powered using a small signal generation device and a dry battery; it demonstrated it could conduct the dynamic co-culture medium perfusion for a liver and breast cancer model and ensured the enhanced cell growth, viability, and function reproduction compared with the static control group.

Magnetorheological elastomer (MRE) is a composite material made of a nonmagnetic polymeric matrix and ferromagnetic particles. The encapsulated ferromagnetic particles mean the material is affected by magnetic fields and can be made to act as a pump in certain configurations with a changing magnetic field. Based on this innovative actuation principle, Tang et al. proposed integrated magnetorheological elastomer microactuators for on-chip pump and mixing functionality without delicate mechanical and electronic components, providing great benefit for fabrication of portable and compact OOC systems [96,97]. Generally, extreme strains and stresses are not needed to induce mechanical stimuli to on-chip organs for reconstructing dynamic microenvironments in human. Therefore, most alternative actuating systems can produce sufficient levels of mechanical stimuli. However, the fabrication of miniaturized actuators with batch-to-batch consistency, the development of corresponding control systems with fine-tuned parameters, and the precise generation of proper levels of mechanical stimuli without using feedback control provided by sophisticated computer-operated systems could be challenging.

Hydrogel material has been used to fabricate the scaffold for cell growth and has shown great cellular compatibility for the prolonged culture and customizable deformability based on mimicking organ features. The usage of natural and synthetic hydrogels has been extensively explored and used in microfluidic applications serving as the actuators because the material is sensitive to extra applied fields like magnetic field and is able to convert to physical deformation in response, which means hydrogels could be an effective alternative to current pneumatic systems [98,99,100,101]. On the other hand, it should be noted that hydrogel actuators require specific environments (such as changing pH and temperature) to operate, which adds extra parameters (and maybe sources of uncertainty) to be controlled. This may limit the potential of hydrogel actuators, as they need to be carefully designed to avoid the introduction of undesirable influence on the organ microenvironment.

While different types of microfluidic OOC platforms have been designed and fabricated for proof-of-concept research, progress of transformation from lab study to commercial products is still slow. The major targeted end-users of OOC devices are researchers in biological, biomedical, and pharmaceutical fields, who may lack sufficient engineering skills. Nonetheless, complex setup configurations and rather complicated fabrication processes for current devices impede the popularization of OOC to replace conventional culture plates. In addition to engineering problems, another major issue of OOC technology is that the organ-level functional replication is limited by the source of cells. This is mainly caused by the fact that most OOC platforms are unable to expand primary cells and therefore need to establish cell cultures directly from donors or patients. It is also impossible to fully simulate the complex conditions and structures of human organ physiological functions simply using cell-based studies. To this end, the development of OOC technology is still in its infancy.
